# B Chromosomes of *Aegilops speltoides* Are Enriched in Organelle Genome-Derived Sequences

**DOI:** 10.1371/journal.pone.0090214

**Published:** 2014-02-26

**Authors:** Alevtina Ruban, Jörg Fuchs, André Marques, Veit Schubert, Alexander Soloviev, Olga Raskina, Ekaterina Badaeva, Andreas Houben

**Affiliations:** 1 Russian State Agrarian University – Moscow Timiryazev Agricultural Academy, Department of Genetics and Biotechnology, Moscow, Russia; 2 Leibniz-Institute of Plant Genetics and Crop Plant Research (IPK), Chromosome Structure and Function Laboratory, Gatersleben, Germany; 3 Laboratory of Plant Cytogenetics and Evolution, Department of Botany, Universidade Federal de Pernambuco, Recife, Brazil; 4 Institute of Evolution, University of Haifa, Laboratory of Plant Molecular Cytogenetics, Haifa, Israel; 5 Engelhardt Institute of Molecular Biology Russian Academy of Sciences, Laboratory of Molecular Karyology, Moscow, Russia; Ben-Gurion University, Israel

## Abstract

B chromosomes (Bs) are dispensable components of the genome exhibiting non-Mendelian inheritance. Chromosome counts and flow cytometric analysis of the grass species *Aegilops speltoides* revealed a tissue-type specific distribution of the roughly 570 Mbp large B chromosomes. To address the question whether organelle-to-nucleus DNA transfer is a mechanism that drives the evolution of Bs, *in situ* hybridization was performed with labelled organellar DNA. The observed B-specific accumulation of chloroplast- and mitochondria-derived sequences suggests a reduced selection against the insertion of organellar DNA in supernumerary chromosomes. The distribution of B-localised organellar-derived sequences and other sequences differs between genotypes of different geographical origins.

## Introduction

B chromosomes (Bs) are optional additions to the basic set of standard A chromosomes (As), and occur in all eukaryotic groups. They differ from the As in inheritance, and Bs are not required for normal growth and development of the host organism. Due to the dispensable nature of Bs, they can be present or absent among individuals of the same population in a species. It is widely accepted that B chromosomes derived from the A chromosomes and/or from sex chromosomes. However, there is also evidence suggesting that Bs can be spontaneously generated in response to the new genome conditions following interspecific hybridisation (for reviews, see [1]–[3]).

Sequence characterisation of the B chromosome of rye provided a unique opportunity for the analysis of the origin and evolution of this enigmatic genome component [4], [5]. In contrast to the prevalent view that Bs do not harbor genic sequences, analyses showed that rye Bs are rich in partly transcriptionally active gene-derived sequences [6]. This allowed us to trace their origin from duplicated fragments of multiple A chromosomes [4]. Beside the amplification of B-located satellite repeats [5], this selfish chromosome has accumulated significantly greater amounts of chloroplast- and of mitochondrion-derived sequences than the A chromosomes [4]. Almost all parts of the chloroplast and mitochondrial genomes are found on the Bs, indicating that all sequences are transferable. The higher amount of organelle-derived DNA inserts in B than in A chromosomes and an increased mutation frequency of B-located organellar DNA suggests a reduced selection against the insertion of organellar DNA in supernumerary chromosomes. To study whether the in rye observed B-specific accumulation of organelle-derived DNA can also be found in other species we analysed the B chromosomes of the grass *Aegilops speltoides*.

*Aegilops speltoides* Tausch (syn. *Triticum speltoides* (Tausch) Gren.) is an annual diploid species (2n = 2x = 14, genome type: S) which belongs to section *Sitopsis* (Triticeae, Poaceae). This species is mainly distributed in the Fertile Crescent and also occurs in south-eastern part of the Balkan peninsula [7]. The submetacentric Bs of *Ae. speltoides* are about 2/3 of the average length of the A chromosomes [8]. The Bs of this species are absent in the roots but stably present in the aerial tissue of the same individual [9], and a maximum number of eight Bs per cell was reported [10]. The maintenance of Bs in natural populations is possible by their transmission at higher than Mendelian frequencies. Accumulation of Bs occurs due to the non-disjunction of B chromatids and the preferential transport of both B chromatids into the generative nucleus during the first mitosis in the male gametophyte [9]. *Aegilops* Bs probably originated from the standard set of As as a consequence of interspecific hybridisation and/or chromosomal rearrangement events. Proposed potential donors of the Bs are the A chromosomes 1, 4 and 5 of the *Ae. speltoides* genome [11], [12]. Consequently, the Bs are also characterized by a number of A chromosome-localised repeats like *Spelt1*, pSc119.2 tandem repeats, 5S rDNA and *Ty3-gypsy* retroelements [11]–[14].

## Materials and Methods

### Plant material and plant cultivation

Plants from natural populations of *Aegilops speltoides* ssp. *aucheri* (Boiss.) Chennav. from Katzir, Israel (TS 89 Mediterranean, 233–250 m, 32829′N, 35805′E), Technion, Haifa, Israel (2.36 Mediterranean, 224 m, 32846′N, 35800′E), Ramat Hanadiv, Israel, (2.46 Mediterranean, 100–125 m, 32833′N, 34856′E) and Tartus, Syria (PI 487238 Mediterranean, 600 m, 35807′N, 36807′E) were cultivated first under greenhouse conditions (16 h light, 22°C day/16°C night) and were further cultivated in a garden (Gatersleben, Germany) under natural condition. The plant material was provided by USDA, Aegean Agricultural Research Institute (Turkey) and the Institute of Evolution, Haifa (Israel).

### Flow cytometric genome size determination

The genome size was determined using a CyFlow Space (Partec) or a FACSAria Flow Cytometer (BD Biosciences) following the protocol of Dolezel et al. [15]. The analysis is based on the mean of five independent measurements of individuals with known number of Bs. For all measurements, *Secale cereale* L. (Genebank Gatersleben, accession number R 737) with an estimated genome size of 16.19 pg/2C was taken as internal reference standard.

### Chromosome preparations derived from shoot meristems

Young secondary shoots before stem elongation were used for chromosome preparation. After an ice-water treatment of 24 hours the material was fixed in 3∶1 (ethanol:acetic acid) and stored at 4°C until use from 4 days to several weeks. The fixative was changed after first hour of fixation and then each few weeks during storage. The basal parts of shoots were excised and washed first in ice-cold water and next twice in 1× citric buffer. Meristematic tissue was treated with an enzyme mixture (0.7% cellulase R10, 0.7% cellulase, 1.0% pectolyase, and 1.0% cytohelicase in 1× citric buffer) for 50 min at 37°C. Afterwards material was washed in 1× citric buffer and twice in ice-cold water. The shoot meristem was fragmented in 7 µl of 60% freshly prepared acetic acid into smaller pieces with the help of a needle on a slide. After, another 7 µl of 60% acetic acid was added the specimen was kept for 2 min at room temperature. Next, a homogenization step was performed with an additional 7 µl 60% acetic acid and the slide was placed on a 50°C hot plate for 2 min. The material was spread by hovering a needle over the drop without touching the hot slide. After spreading of cells, the drop was surrounded by 200 µl of ice-cold, freshly prepared 3∶1 fixative. More fixative was added and the slide was shortly washed in fixative, then dipped in 60% acetic acid for 10 min and rinsed 5 times in 96% ethanol. A quality check of the air dried slides was performed by phase contrast microscopy. The slides were stored until use in 96% ethanol at 4°C.

### Fluorescence *in situ* hybridisation (FISH) and microscopy

The following probes were used: barley BACs encoding chloroplast DNA (BAC clone ChHB 100G01) and mitochondrial DNA (BAC clone MnHB 0205G01) [4], [5] and the *Arabidopsis*-type telomere repeat [16]. Amplicons for the 5S ribosomal DNA, which include the coding as well as the flanking spacer region, were generated by PCR as described by Fukui et al. [17]. The *Spelt-1* tandem repeat probe was generated by PCR according to Salina et al. [18]. FISH probes were labelled with ChromaTide Texas Red-12-dUTP or Alexa Fluor 488-5-dUTP (http://www.invitrogen.com) by nick translation. FISH was performed as described by Ma et al. [16]. Fluorescence signals were observed by standard epifluorescence microscopy or to achieve a resolution of ∼100 nm by structure illumination microscopy (SIM) using an Elyra super resolution microscope (Zeiss). All images were collected in grey scale and pseudocoloured.

## Results and Discussion

### Tissue type-specific distribution of B chromosomes

**Figure 1:**
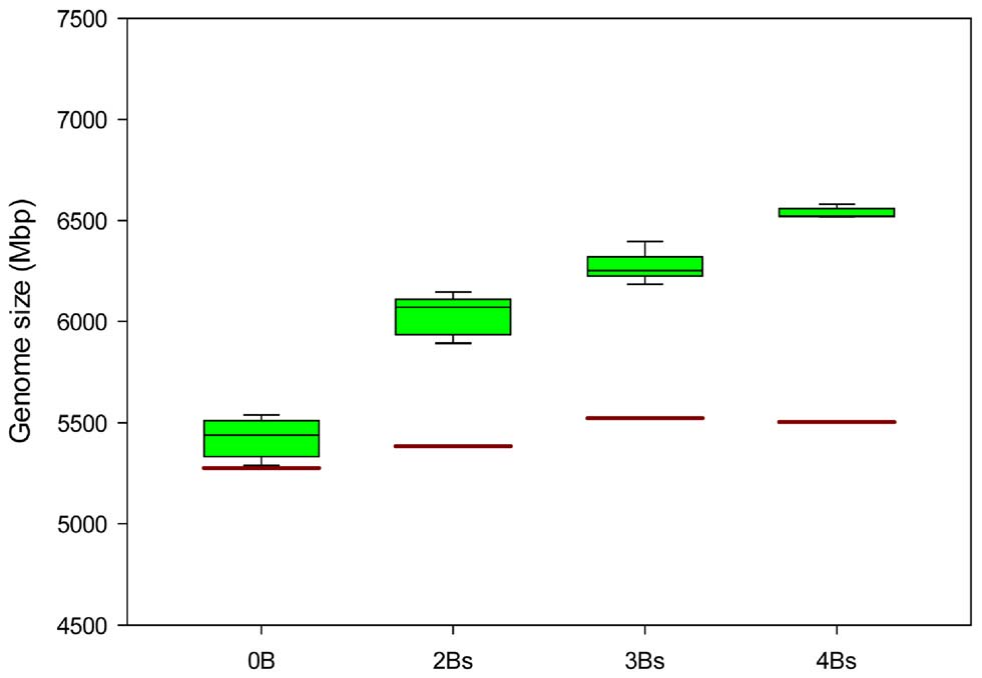
Size determination of the B chromosome. Boxplot representing the genome size distribution in plants without and with B chromosomes of *Ae. speltoides* from Katzir (Israel) measured by flow cytometry. In plants without Bs no difference was found between nuclei isolated from leaf (green boxes) and root (brown line) tissues, while it differed remarkably in plants with Bs. The box boundaries indicate the 75^th^ and 25^th^ percentiles and the error bars the 90^th^ and 10^th^ percentiles of the five replicates.

Since B chromosomes of *Ae. speltoides* are known to possess 5S rDNA sites, plants carrying B chromosomes were screened based on additional hybridisation signals after FISH on interphase nuclei of leaf tissue using a corresponding hybridisation probe. This allowed an unambiguous identification of B-positive plants at an early stage of development as the Bs of this species are absent in roots (Figure S1). The tissue-type specific distribution pattern of Bs was confirmed by flow cytometric analysis of 0B, 2B, 3B and 4B plants from the Katzir (Israel) population (Table S1). The average size of a single unreplicated B chromosome was estimated to be ∼570 Mbp, ranging from 550 to 600 Mbp, for (1C). Hence, one B equals ∼10% of the genome size of a 0B plants (5,400 Mbp). The genome size of nuclei isolated from roots was similar in all analysed plants, while it increased in leaf nuclei depending on the number of Bs (Figure 1). An analysis of Bs in differentiated tissues has not been reported before. A comparable situation of tissue-type specific B chromosome distribution is also known for species as, *Agropyron cristatum* [19], *Poa alpina* [20], or *Aegilops mutica* [21]. The absence of Bs in some organs or tissues could be caused by a specific elimination process of Bs during an early stage of embryo differentiation.

### The Bs are polymorphic and enriched in organellar -derived DNA sequences

**Figure 2:**
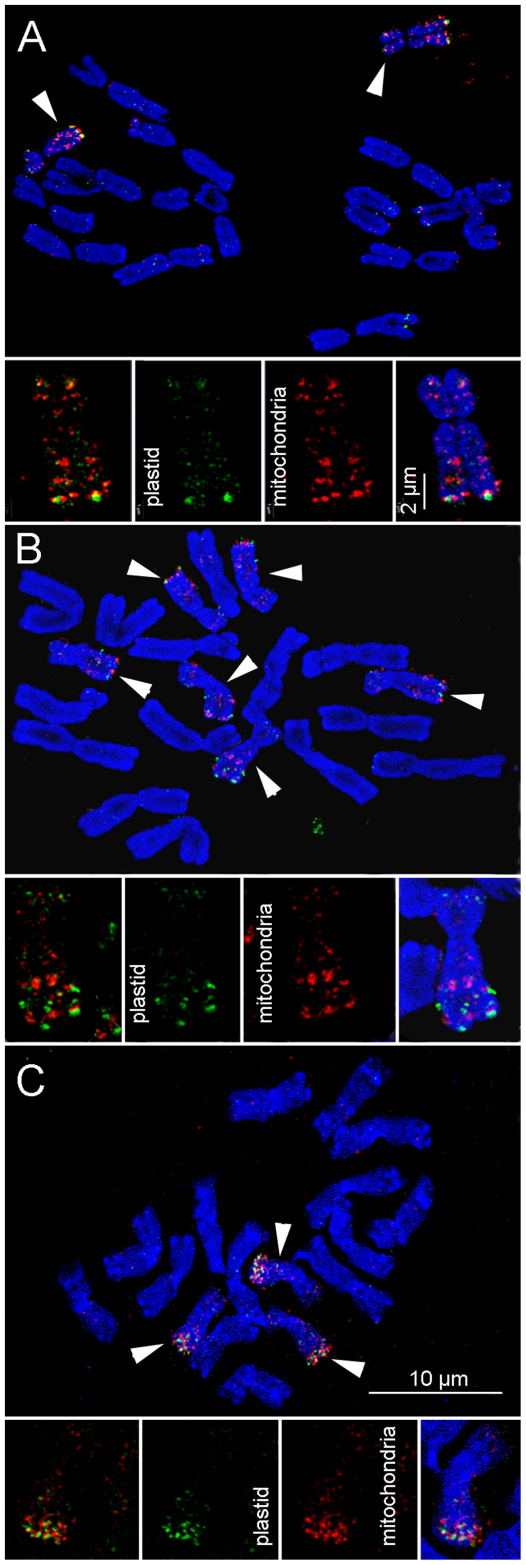
B chromosomes of *Aegilops speltoides* are enriched in organelle genome-derived sequences. Distinct chromosomal localisation of plastid (in green) and mitochondria (in red) derived sequences in three *Ae. speltoides* populations after applying SIM. (A) Somatic metaphase cells with 2 Bs from Katzir (Israel), (B) with 6 Bs from Tartus (Syria), and (C) with 3 Bs from the Technion (Israel) population. Note the geographical origin-dependent hybridisation patters along the Bs (arrowed).

To determine whether the Bs of *Ae. speltoides* from Katzir accumulated mitochondria- and plastid-derived sequences as demonstrated for the Bs of rye [4], we hybridised labelled BACs encoding barley plastid and mitochondrial DNA with *Ae. speltoides* mitotic chromosomes. Both types of probes revealed B-enriched hybridisation signals (Figure 2A). Multiple mitochondria-derived DNA insertions were found along both arms of the B chromosomes except at the pericentromere. The global distribution of plastid DNA-specific signals was similar but of less amount, likely due to a lower abundance. The A chromosomes revealed only minor insertions of mitochondrial origin mainly (Figure S2).

To address whether Bs present in geographically distinct population of *Ae. speltodies* are polymorphic we analysed the chromosomal distribution patterns of organellar DNA in Bs derived from different accessions. As B chromosomes are dispensable, it is expected to observe polymorphisms among populations. The distribution of B-localised organellar-derived signals differed between the tested genotypes. Bs from Tartus showed less organellar-derived signals, although exhibiting a similar distribution as for the Bs from Katzir (Figure 2B). In contrast, Bs from the Technion population displayed a strong accumulation in the subtelomeric region of the long arm for both types of probes (Figure 2C). A partial colocalisation of both mitochondria and plastid DNA-specific signals was found in all Bs independently of their geographical origin. In contrast, a comparative analysis of rye Bs coming from geographically distinct populations did not identify polymorphisms regarding the distribution of accumulated organellar DNA and different B-specific repeats [22]. We conclude that the Bs of *Ae. speltoides* accumulated a significant amount of organellar DNA and that differences of the molecular composition exist between the Bs of different geographical origin. These differences between populations are also reflected by the distribution of FISH signals in interphase nuclei (Figure S3).

**Figure 3:**
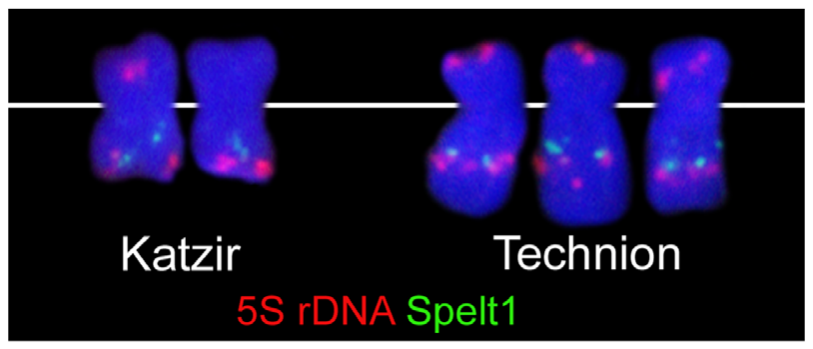
Localisation of 5S rDNA and *Spelt-1* tandem repeat along selected Bs. Deficient-B type from the Katzir population and standard Bs from the Technion population.

The observed polymorphism was further tested by applying the *Triticeae*-specific tandem repeat *Spelt-1*, which is a dynamic component of the *Ae. speltoides* genome [11], [18], as well as of the 5S rDNA. Bs of the Technion and Katzir population revealed 5S rDNA signals localised in the terminal part of short arm and in the long arm near the *Spelt-1* cluster. In some plants of the Katzir population the 5S rDNA locus in the short arm is absent (Figure 3). *Ae. speltoides* Bs are submetacentric, but in one plant from the Katzir population we found a metacentric type of Bs. Likely due to terminal deletion in the long arm, former interstitial 5S rDNA and *Spelt-1* clusters became terminal (Figure 3). Apparently, the repeat clusters of the B are hot spots of chromosomal rearrangements. Comparable intraspecific differences in the patterns of repeat clusters were reported previously for the A chromosomes of *Ae. speltoides* [13]. The localisation of *Arabidopsis*-type telomeric repeats is identical on the A- and B-chromosomes of all populations analysed (Figure S4).

What mechanism could account for the accumulation of organellar DNA on the Bs of *Ae. speltoides*? The first possibility is that insertion into B chromosome DNA has fewer deleterious genetic consequences than their counterparts on As. Insertions into A chromosomes may disrupt gene function with lethal consequences. In contrast, Bs which are not required for growth and development can tolerate more mutations. The second possibility is that the nuclear integration of organellar sequences may be dependent on the formation of double strand breaks and if Bs are particularly prone to double strand breaks, this could facilitate the preferred integration of promiscuous DNA in Bs.

Plastid DNA fragments are very numerous in some tissues such as the developing pollen gametophyte [23] or after stress [24]. Hence, uncontrolled insertion would be expected to result in the accumulation of plastid- and, by analogy, mitochondria-derived sequences. Alternatively, the mechanisms that prevent nuclear genome expansion may be impaired on the Bs. Transfer of organellar DNA to the nucleus is very frequent [25], but much of the promiscuous DNA also is rapidly lost again within one generation [26]. If this elimination mechanism (e.g. illegitimate recombination) is impaired in Bs, the high turnover rates that prevent such sequences from accumulating on the A chromosomes would be absent. Thus, the dynamic equilibrium between frequent integration and rapid elimination of organellar DNA could be impaired for B chromosomes. Alternatively, a B-specific amplification process increased the copy number of integrated organellar DNA.

The observed partial overlapping of plastid and mitochondria DNA-specific hybridisation patterns suggests that both types of promiscuous DNA are chromosomally inserted under similar constrains in *Ae. speltoides* Bs. Future analyses of other B-bearing species such as maize will be needed to address the question whether organelle-to-nucleus DNA transfer is an important mechanism that drives the evolution of B chromosomes [27].

## Supporting Information

Figure S1 **FISH of isolated** ***Ae. speltoides*** **nuclei labelled with 5S rDNA.** (A) Nucleus of a plant without Bs and (B) with Bs (arrowed). (B) The large 5S rDNA signals are of A chromosome origin, while the arrowed minor signals are B chromosome derived. Scale bar equals 10 µm.
(TIF)

Figure S2 **Localisation of mitochondrial- and plastid- derived sequences on** ***Ae. speltoides*** **metaphase chromosomes of a plant without Bs from the Ramat Hanadiv population.** Scale bar equals 10 µm.
(TIF)

Figure S3 **Localisation of mitochondria (in red)- and plastid (in green) derived sequences on** ***Ae. speltoides*** **interphase nuclei.** (A) Nucleus of a plant with 2Bs from Katzir. (B) Nucleus of a plant with 3Bs from the Technion population. Scale bar equals 10 µm.
(TIF)

Figure S4 **Localisation of** ***Spelt-1*** **tandem repeat (in green) and** ***Arabidopsis*** **-type telomeric repeat (in red) sequences on** ***Ae. speltoides*** **metaphase chromosomes.** The Bs are marked with arrows. Scale bar equals 10 µm.
(TIF)

Table S1 **Genome size determination of** ***Ae. speltoides*** **plants with and without Bs. Flow cytometry was used to determine the genome size of nuclei isolated from leaf tissue.**
(DOCX)
