# Supplementary figures and images for: B Chromosomes of Aegilops speltoides Are Enriched in Organelle Genome-Derived Sequences
Source: PLoS One. 2014 Feb 26;9(2):e90214. doi: 10.1371/journal.pone.0090214 (PMC3936023; doi:10.1371/journal.pone.0090214)

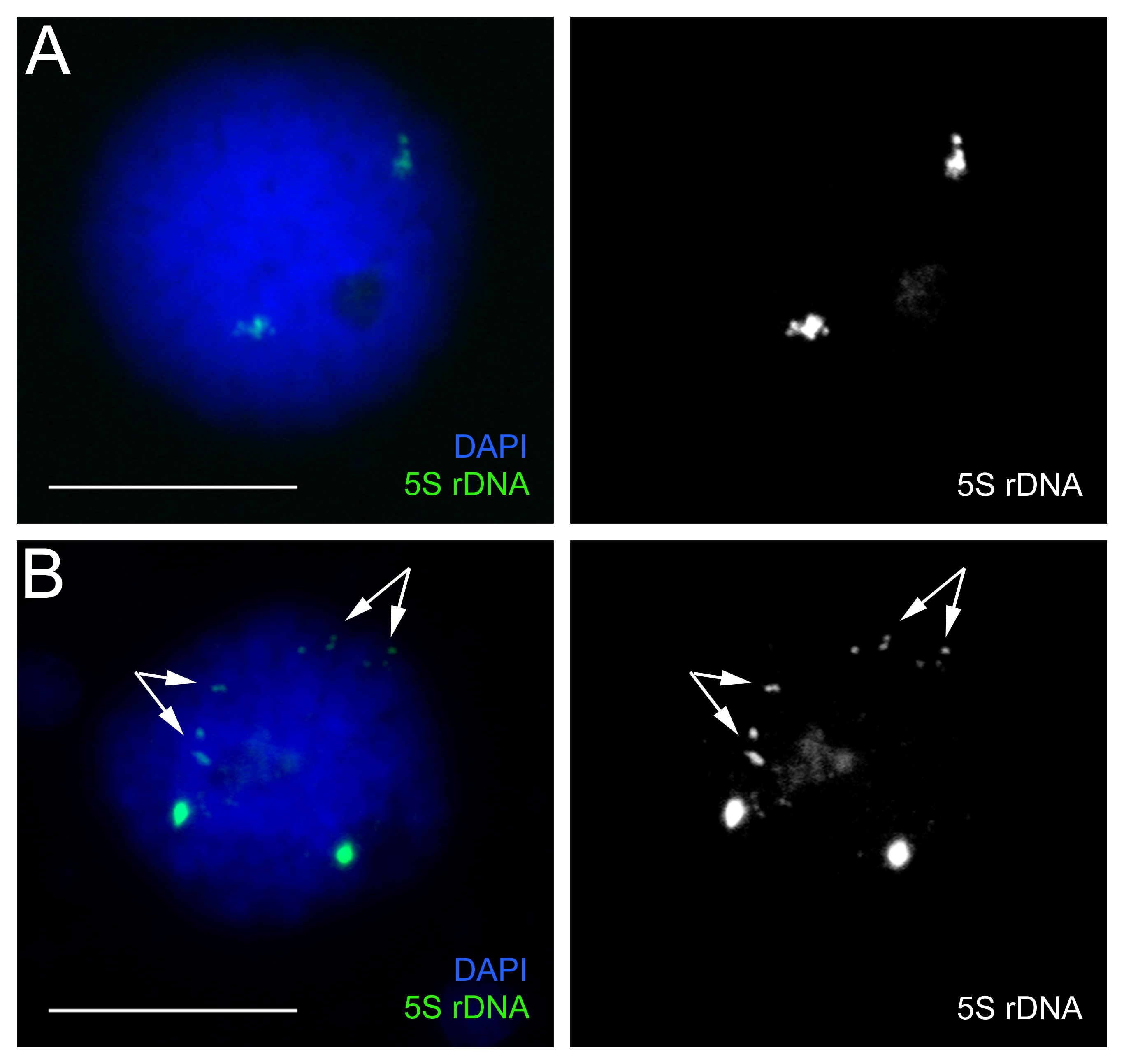

Supplement: Figure S1 — FISH of isolated Ae. speltoides nuclei labelled with 5S rDNA. (A) Nucleus of a plant without Bs and (B) with Bs (arrowed). (B) The large 5S rDNA signals are of A chromosome origin, while the arrowed minor signals are B chromosome derived. Scale bar equals 10 µm. (TIF) [file pone.0090214.s001.tif]

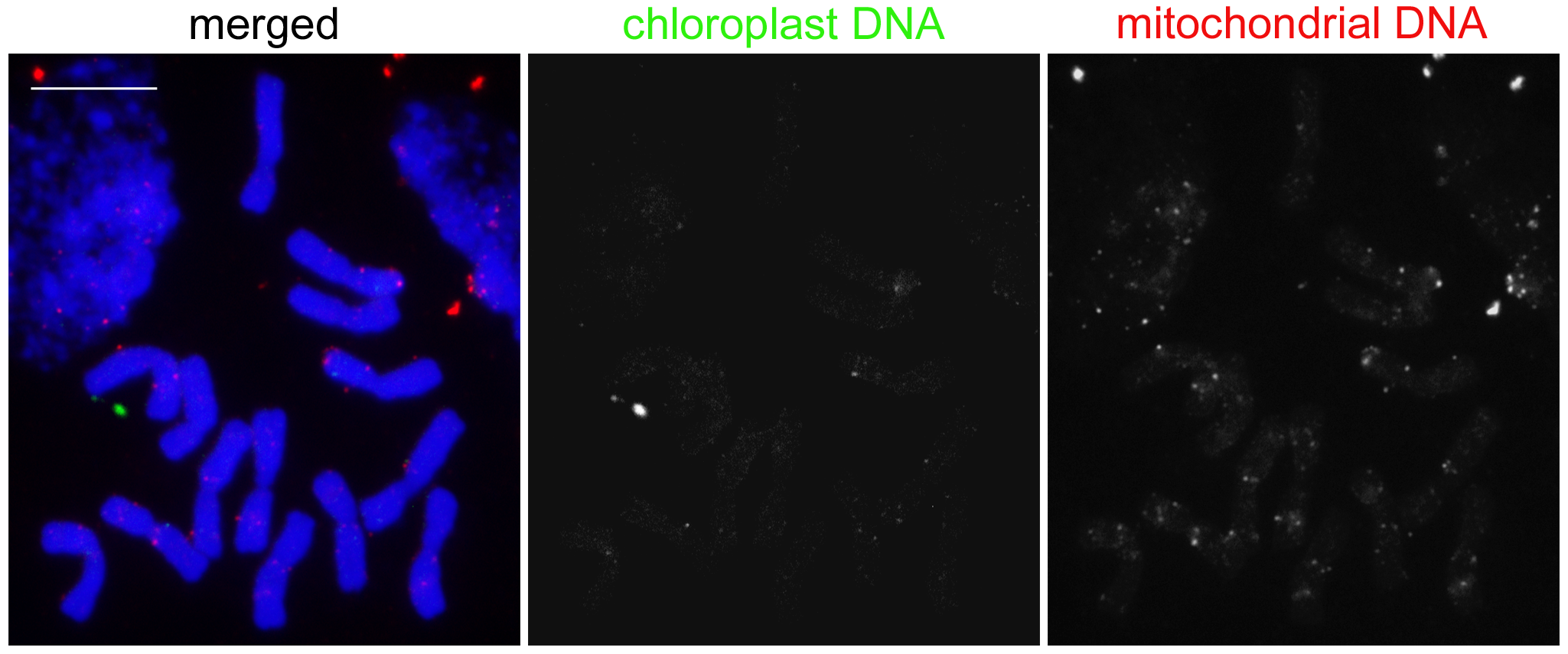

Supplement: Figure S2 — Localisation of mitochondrial- and plastid- derived sequences on Ae. speltoides metaphase chromosomes of a plant without Bs from the Ramat Hanadiv population. Scale bar equals 10 µm. (TIF) [file pone.0090214.s002.tif]

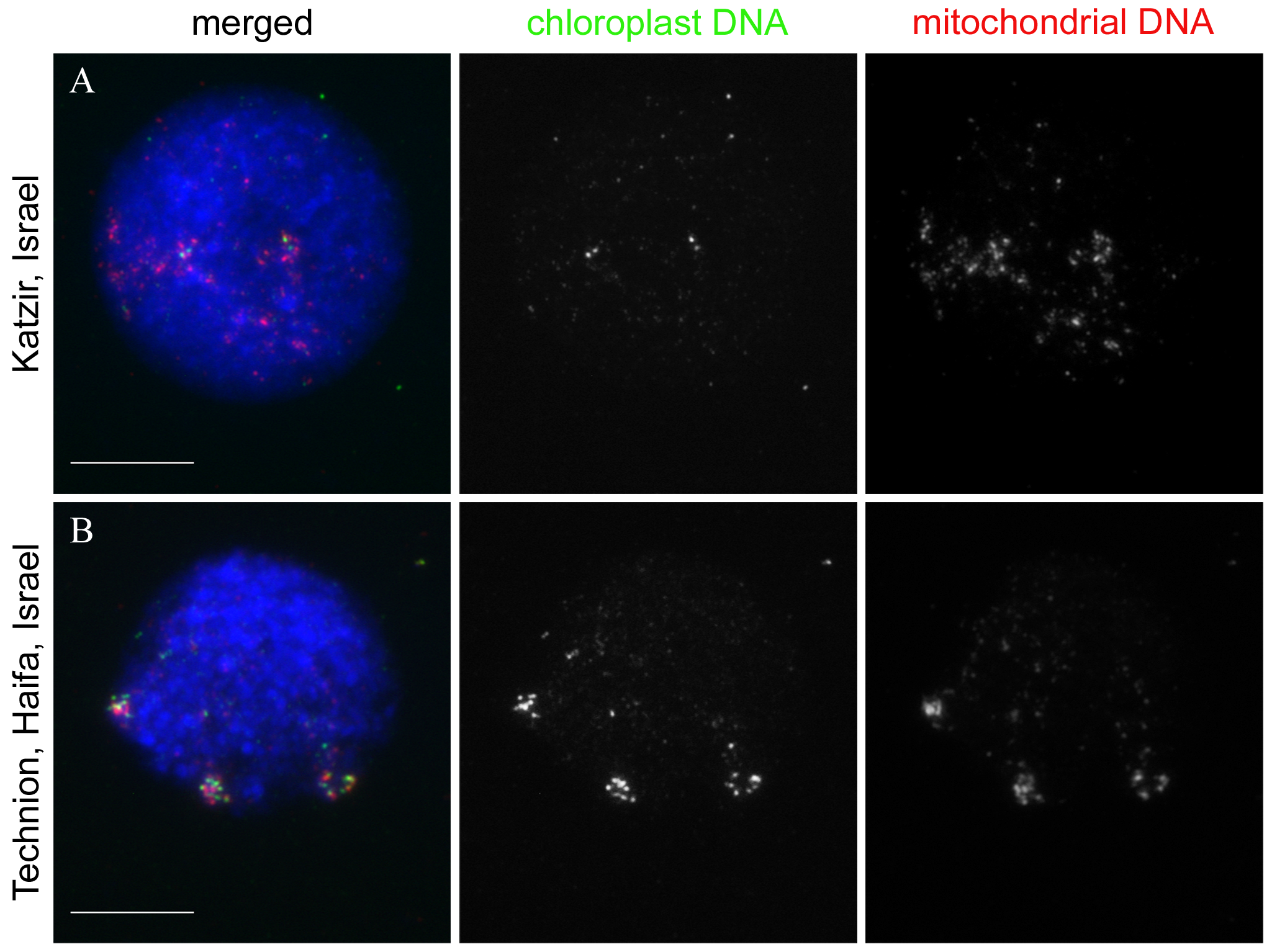

Supplement: Figure S3 — Localisation of mitochondria (in red)- and plastid (in green) derived sequences on Ae. speltoides interphase nuclei. (A) Nucleus of a plant with 2Bs from Katzir. (B) Nucleus of a plant with 3Bs from the Technion population. Scale bar equals 10 µm. (TIF) [file pone.0090214.s003.tif]

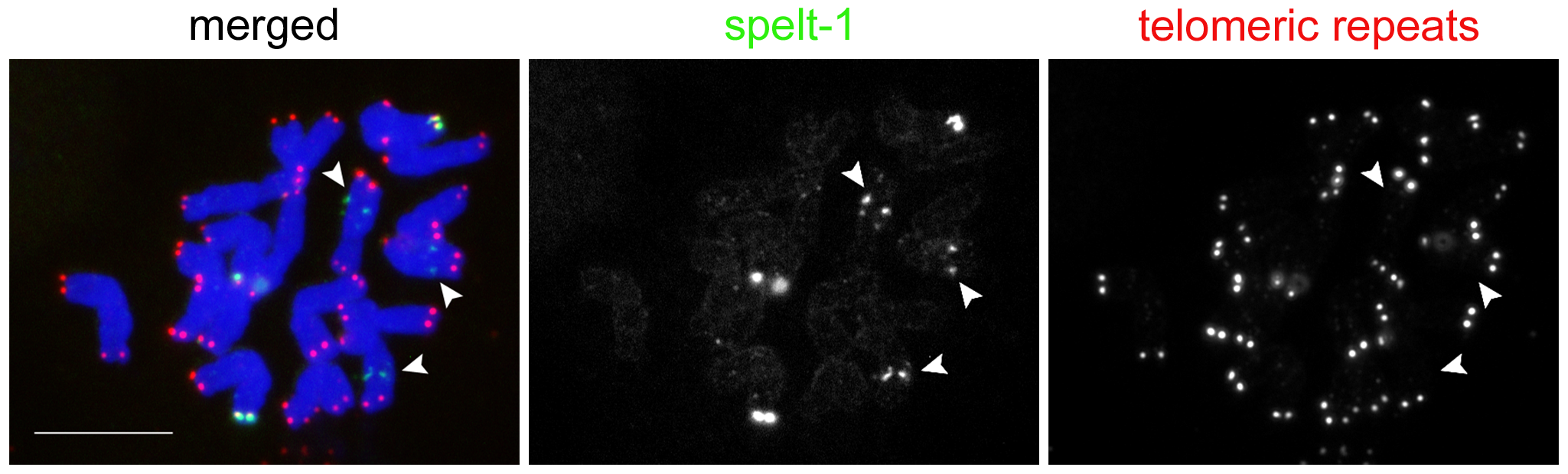

Supplement: Figure S4 — Localisation of Spelt-1 tandem repeat (in green) and Arabidopsis -type telomeric repeat (in red) sequences on Ae. speltoides metaphase chromosomes. The Bs are marked with arrows. Scale bar equals 10 µm. (TIF) [file pone.0090214.s004.tif]
