# Supplementary material for: B Chromosomes of Aegilops speltoides Are Enriched in Organelle Genome-Derived Sequences
Source: PLoS One. 2014 Feb 26;9(2):e90214. doi: 10.1371/journal.pone.0090214 (PMC3936023; doi:10.1371/journal.pone.0090214)
Supplement: Table S1 — Genome size determination of Ae. speltoides plants with and without Bs. Flow cytometry was used to determine the genome size of nuclei isolated from leaf tissue. (DOCX) [file pone.0090214.s005.docx]

Table S1. Genome size determination of *Ae. speltoides* plants with and without Bs. Flow cytometry was used to determine the genome size of nuclei isolated from leaf tissue.

| Number of Bs | Replicate | Nuclear DNA content, pg/2C | Average | Difference | Gain in Mbp | Average size of Bs, Mbp±SEM | Size of single unreplicated B, Mbp±SEM* |
| --- | --- | --- | --- | --- | --- | --- | --- |
| 0B | 1 | 11.25 | 11.09 | - | - | - | - |
|  | 2 | 10.82 |  | - | - | - | - |
|  | 3 | 11.12 |  | - | - | - | - |
|  | 4 | 10.94 |  | - | - | - | - |
|  | 5 | 11.33 |  | - | - | - | - |
| 2Bs | 1 | 12.16 | - | 1,07 | 1051 | 1216±95 | 608±48 |
|  | 2 | 12.05 | - | 0,96 | 940 |  |  |
|  | 3 | 12.41 | - | 1,32 | 1295 |  |  |
|  | 4 | 12.57 | - | 1,48 | 1445 |  |  |
|  | 5 | 12.47 | - | 1,38 | 1352 |  |  |
| 3Bs | 1 | 12.65 | - | 1,56 | 1522 | 1700±71 | 567±24 |
|  | 2 | 13.08 | - | 1,99 | 1946 |  |  |
|  | 3 | 12.76 | - | 1,67 | 1630 |  |  |
|  | 4 | 12.79 | - | 1,70 | 1659 |  |  |
|  | 5 | 12.88 | - | 1,79 | 1746 |  |  |
| 4Bs | 1 | 13.34 | - | 2,25 | 2196 | 2232±24 | 558±6 |
|  | 2 | 13.34 | - | 2,25 | 2199 |  |  |
|  | 3 | 13.46 | - | 2,37 | 2316 |  |  |
|  | 4 | 13.33 | - | 2,24 | 2189 |  |  |
|  | 5 | 13.4 | - | 2,31 | 2258 |  |  |

* The calculated sizes of single unreplicated B chromosomes are not significantly different between plants harbouring 2 to 4 Bs according to Kruskal-Wallis One Way Analysis of Variance on Ranks (P = 0.827).
